# Advanced alveolar soft part sarcoma responds to apatinib

**DOI:** 10.18632/oncotarget.18599

**Published:** 2017-06-22

**Authors:** Yong Zhou, Fan Tang, Yiying Wang, Li Min, Yi Luo, Wenli Zhang, Rui Shi, Hong Duan, Chongqi Tu

**Affiliations:** ^1^ Department of Orthopedics, West China Hospital, Sichuan University, Chengdu, China; ^2^ Sarcoma Biology Laboratory, Center for Sarcoma and Connective Tissue Oncology, Massachusetts General Hospital, Boston, MA, USA; ^3^ Department of Pathology, West China Hospital, Sichuan University, Chengdu, China

**Keywords:** alveolar soft part sarcoma, vascular endothelial growth factor receptor-2, anti-angiogenesis, chemotherapy resistance, apatinib

## Abstract

Alveolar soft part sarcoma (ASPS) is a rare, hypervascular soft tissue sarcoma with a low chemotherapy response rate. Here, we report an ASPS case with multiple lung metastases on initial presentation. The primary tumor, a hypervascular soft tissue mass 4.1×3.2×2.0 cm, located in the right thigh, was resected prior to chemotherapy. The patient suffered disease progression after two cycles of gemcitabine-docetaxel treatment. Immunohistochemical examination of the tumor tissue revealed strong positive staining for vascular endothelial growth factor (VEGF) and VEGF receptor-2 (VEGFR-2). The patient was subsequently treated with apatinib (500 mg/day), a specific VEGFR-2 inhibitor. Treatment was well tolerated, and the patient exhibited a partial response, with the lung metastases reduced in size and number after one month of therapy. To date, 12-month progression-free survival has been achieved. Apatinib may provide an additional treatment option for metastatic ASPS, particularly in cases resistant to other chemotherapeutic options. Furtherstudies with more cases with longer follow-up times will be necessary to determine the clinical efficacy of apatinib for treatment of ASPS.

## INTRODUCTION

Alveolar soft part sarcoma (ASPS) was first classified by Christopherson, et al. in 1952 [1]. ASPS is a clinically and morphologically unique soft tissue sarcoma that accounts for 0.5-1% of all soft tissue sarcomas, and occurs primarily in teenagers and young adults under 40 years of age [2]. The main ASPS sites are the lower extremities and trunk, and the head and neck, particularly the orbit and tongue [3–5]. ASPS is slow growing and painless and, due to a relative lack of associated symptoms, metastases to the lungs, brain, and liver are common at diagnosis [2, 6]. For early-stage localized ASPS, wide surgical resection is the primary treatment [7]. Local relapse after complete resection is unusual; however, metastases can occur, even decades after primary tumor resection, despite the absence of local recurrence [8]. Although ASPS progression tends to be slow, poor prognosis and resistance to conventional chemotherapeutics pose treatment challenges [9, 10].

Apatinib is a novel receptor tyrosine kinase (RTK) inhibitor that selectively competes for the vascular endothelial growth factor receptor 2 (VEGFR-2) ATP binding site, blocking downstream signaling and inhibiting tumor angiogenesis [11, 12]. The drug was approved by the China Food and Drug Administration in December 2014 for treatment of metastatic gastric cancer patients. Apatinib can improve progression-free survival, and consequently overall survival, in advanced gastric cancer patients [13]. Apatinib could potentially augment therapeutic options in a variety of sarcomas, including angiosarcoma, malignant fibrous histiocytoma, and myxoid/round cell liposarcoma [14–16].

Here, we describe an advanced ASPS case with multiple lung metastases, disease progression after conventional chemotherapy, and subsequent near-complete response to apatinib treatment. We also review the literature and compare the clinical efficacy of apatinib with that of other antiangiogenic therapeutics used to target ASPS. Finally, we discuss the possible mechanisms underlying the ASPS response to apatinib.

## CASE PRESENTATION

This case report was approved by the Medical Ethics Committee of the West China Hospital, and written informed consent was obtained from the patient for publication of this case report and accompanying images.

**Figure 1:**
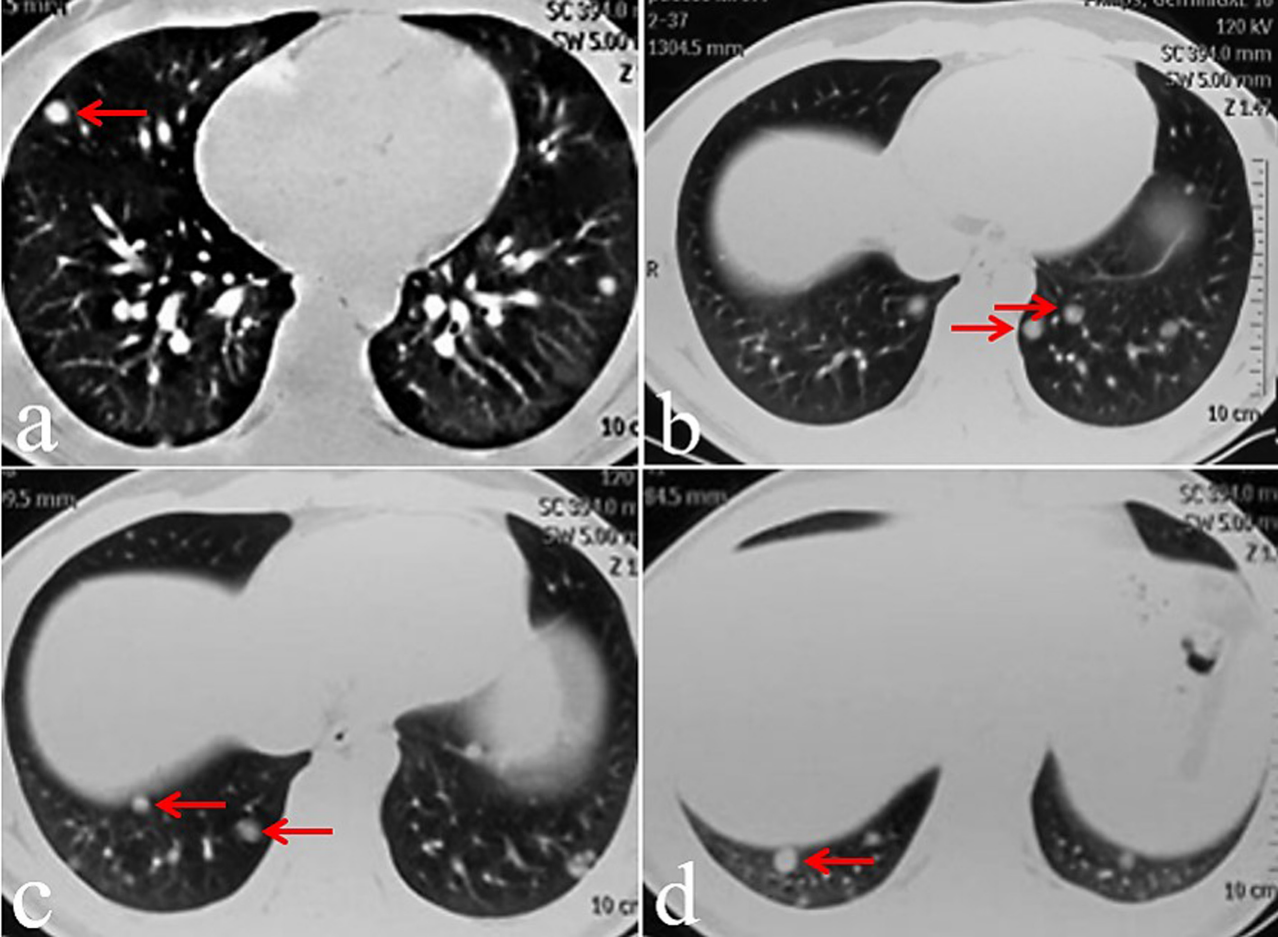
A March 2015 chest CT revealed multiple pulmonary nodules likely representing metastatic disease

**Figure 2:**
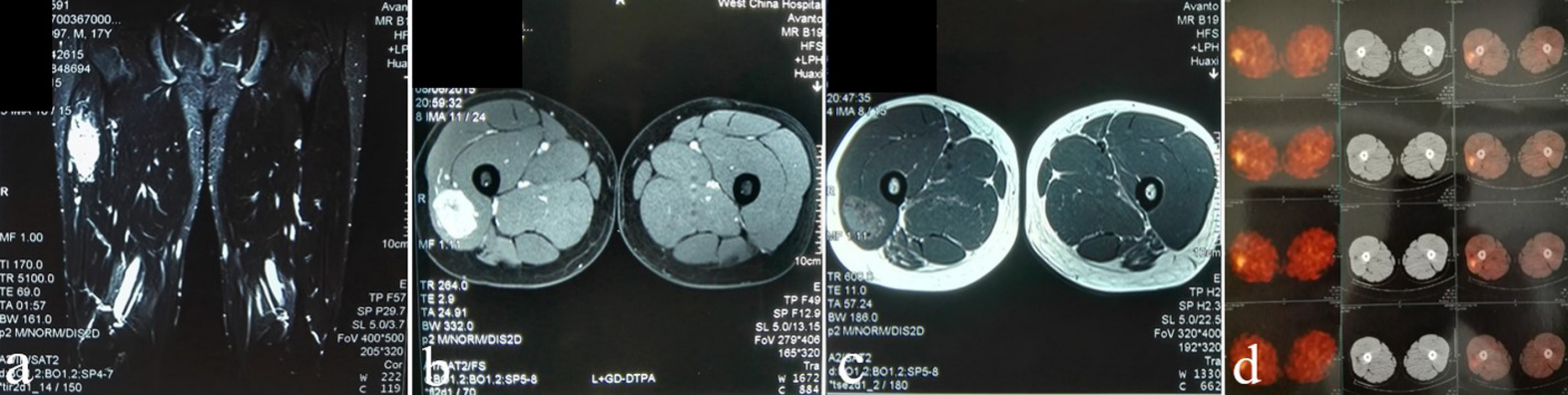
MRI and PET scans A soft tissue is shown in the upper right thigh, with long T1 and T2 signals (**a**-**c**). PET scan showing a soft tissue mass with a maximum SUV score of 2.6 (**d**).

**Figure 3:**
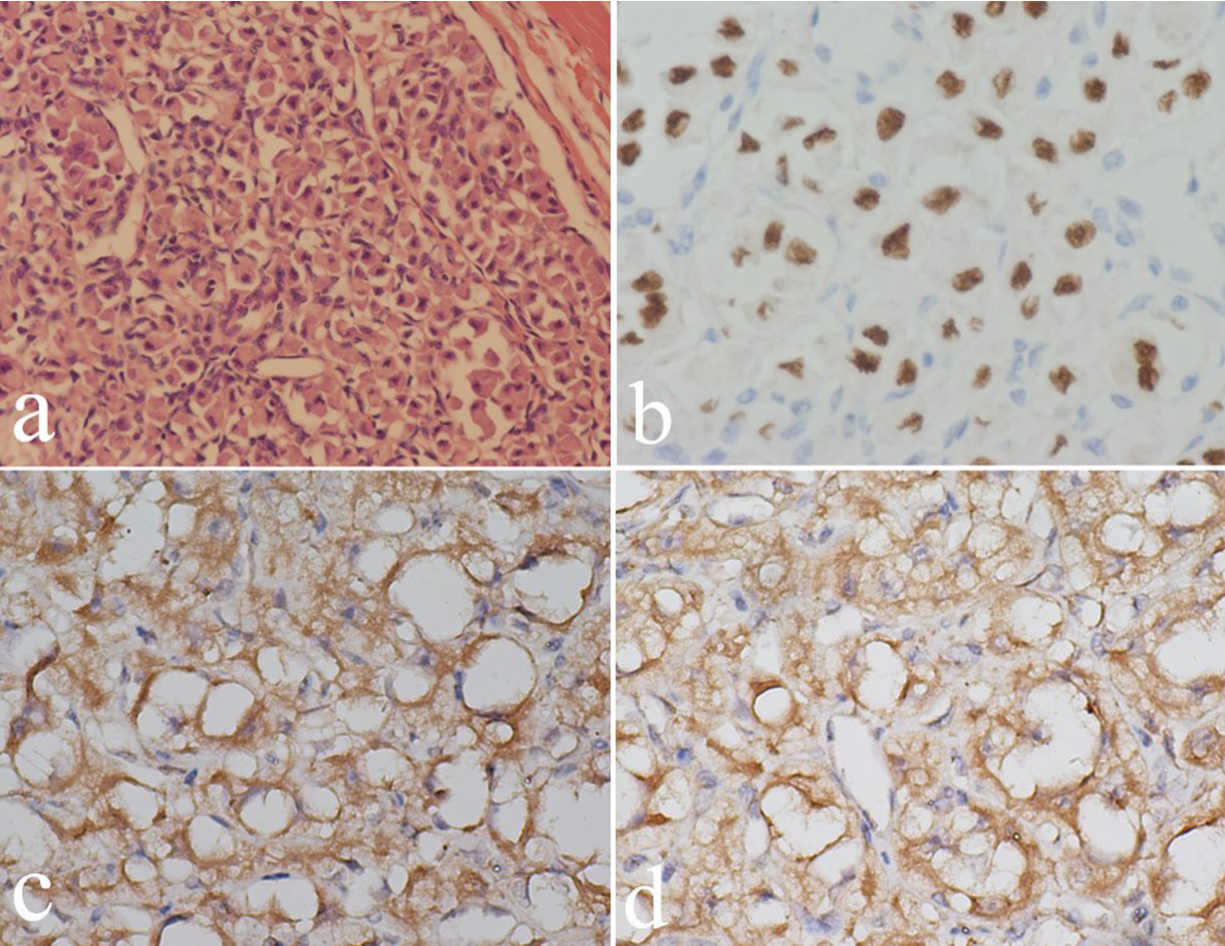
Pathological images of the tumor H&E staining x200) showing solid nests of neoplastic cells separated by delicate fibrovascular septa proliferating in the endometrium (**a**). IHC staining showing TFE3 positive tumor cell nuclei (**b**). IHC staining showing strong VEGF (antibody diluted 1:100) (**c**) and VEGF-2 positivity (antibody diluted 1:200) (**d**).

In March 2015, an 18-year-old male was admitted to the respiratory department of our hospital with a primary complaint of chest tightness. Computed tomography (CT) of the chest revealed multiple pulmonary nodules likely representing metastatic disease (Figure 1). A positron emission tomography (PET) scan to identify the primary tumor revealed a soft tissue mass in the upper right thigh, with a maximum SUV score of 2.6 (Figure 2). Magnetic resonance imaging (MRI) of the thigh revealed a hypervascular soft tissue mass of 4.1 × 3.2 × 2.0 cm (Figure 2). Color Doppler ultrasound of the affected leg confirmed the MRI findings. A mass was of volume, 4.5 × 3.6 × 2.0 cm, was found between the skin and fascia during the extended resection procedure. Pathological examination confirmed ASPS, and immunohistochemistry (IHC) demonstrated that tumor cell nuclei were strongly TEF-3 positive (Figure 3).

**Figure 4:**
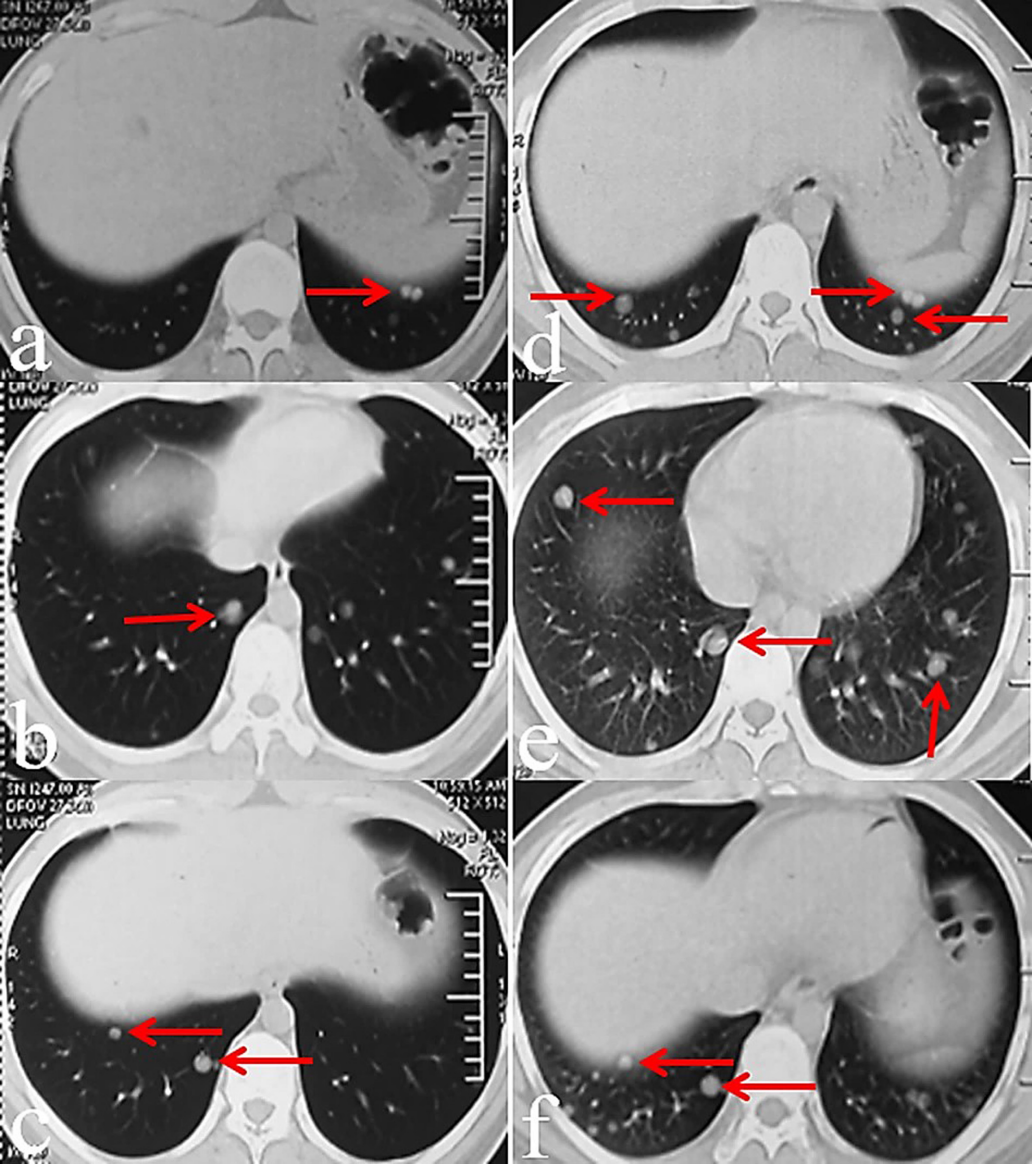
CT images before and after gemcitabine-docetaxel chemotherapy March 2015 CT images collected before gemcitabine-docetaxel chemotherapy, showing multi-lung metastatic nodules (**a**-**c**). May 2015 CT images collected after two gemcitabine-docetaxel chemotherapy cycles, showing disease progression presenting as increased lesion numbers and sizes (red arrows(**d**-**f**).

The patient began to receive gemcitabine-docetaxel chemotherapy two weeks after surgery. Gemcitabine was administered at a fixed dose rate of 900 mg/m2 by intravenous infusion for 90 min on d 1 and 8, with docetaxel (100 mg/m2) administered intravenously for 60 min every 21 d from d 8. In May 2015, after two chemotherapy cycles, chest CT revealed disease progression with additional and larger lung nodules (Figure 4). Considering the observed resistance to gemcitabine-docetaxel chemotherapy, we explored new treatment options for this patient. Additional IHC staining of tumor tissues demonstrated strong VEGF-A and VEGFR-2 positivity (Figure 3), suggesting a possible response to the specific VEGFR-2 inhibitor, apatinib.

**Figure 5:**
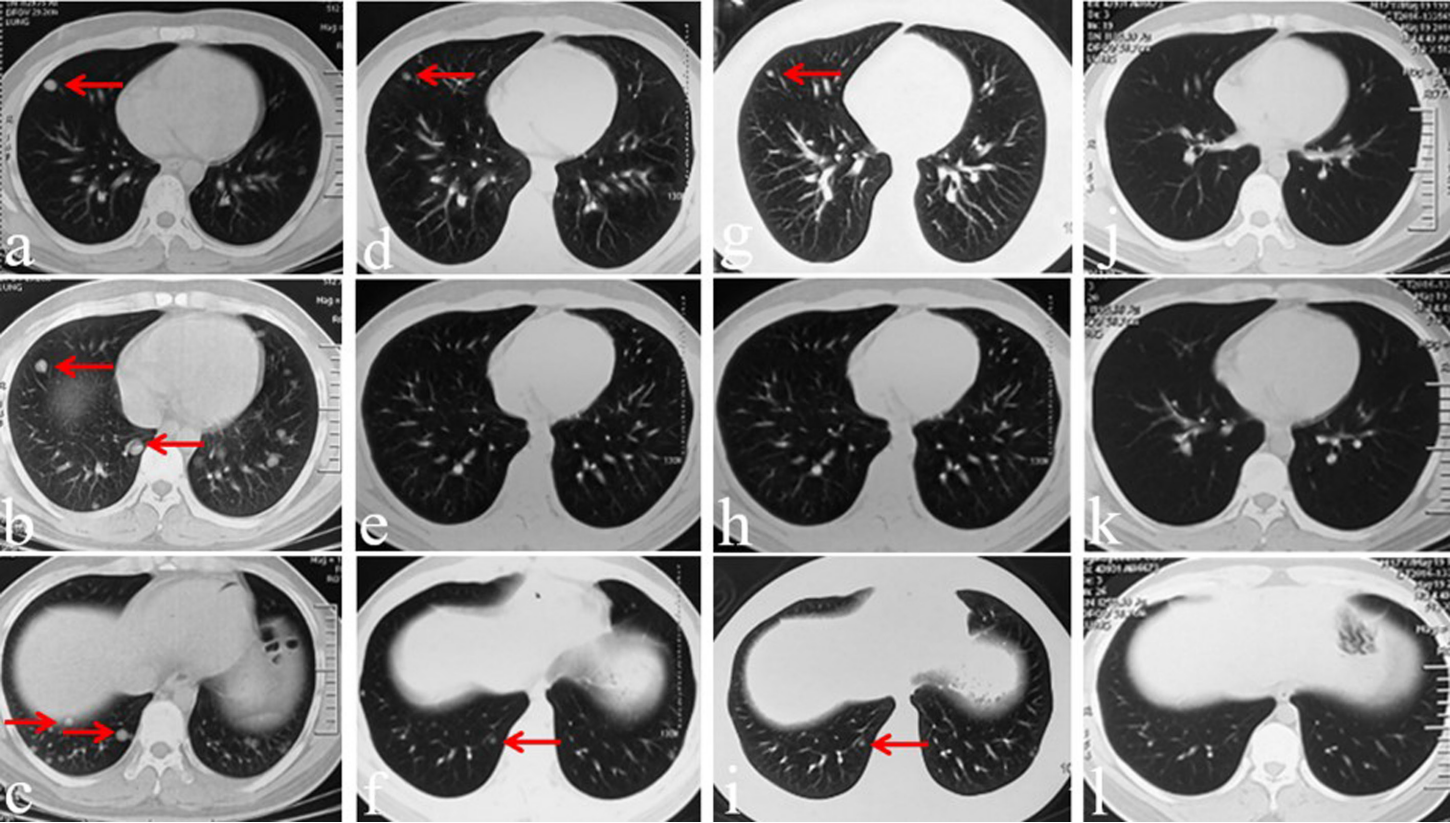
CT images before, during, and after apatinib therapy May 2015 CT images collected before apatinib therapy (**a**-**c**). June 2015 CT images collected one month after apatinib therapy, showing response to treatment presenting as secreased lesion numbers and sizes (**d**-**f**). September 2015 CT images collected three months after apatinib therapy, showing the disappearance of nearly all of the metastatic leasions (**g**-**i**). June 2016 CT images collected three months after apatinib therapy was stopped (**j**-**l**). No disease relapse and no new nodules were observed. No disease relapse and no new nodules were observed.

After the patient provided written, informed consent, we began treatment with apatinib alone in June 2015. Considering his young age and the possible drug toxicities, oral apatinib was administered at a dose of 500 mg/day. After one month, lung metastases were reduced in size and number, with only two small lesions remaining (Figure 5), indicating a partial response according to the Response Evaluation Criteria in Solid Tumors (RECIST) [17]. In September 2015, after three months of treatment, almost all metastatic lesions had disappeared (Figure 5). Apatinib was continued as maintenance therapy for an additional six months and was stopped in March 2016. At the time of this writing, there were no signs of tumor recurrence or new metastatic nodules; 12 months of progression-free survival have been achieved to date. Apatinib-related adverse events experienced by the patient were primarily nonhematological, and included skin rash, short-term elevated alanine transaminase and aspartate amino transferase levels (grade 2, according to the Common Terminology Criteria for Adverse Events v.4.03), and mild hand-foot syndrome (severe toxicity; grades 3-4).

## DISCUSSION

ASPS is a rare, highly vascularized soft tissue sarcoma. Hypervascularity with prominent veins and prolonged capillary staining can be demonstrated through angiography and contrast-enhanced CT scan. ASPS typically presents with long signals on both T2- and T1-weighted MRI images [5]. Histologically, ASPS grows as uniform, organoid nests of polygonal tumor cells with highly vascularized septa (Figure 2). Tumors are characterized by an unbalanced recurrent translocation t(X;17) (p11;q25) leading to expression of the chimeric oncoprotein, ASPL-TFE3 [18], an aberrant transcription factor that promotes ASPS tumorigenesis [19].

Angiogenesis is critical for tumor cell survival, proliferation, local invasion, and metastasis [20, 21]. The roles of VEGF-A, -B, -C, -D, -E, and their receptors (VEGFR-1, VEGFR-2, and VEGFR-3) in tumor angiogenesis, lymphangiogenesis, cell proliferation, and metastasis are well established [22, 23]. VEGFR-2 is the major mediator of known VEGF-induced phenotypes, including microvascular permeability and neovascularization [24]. VEGF/VEGFR-2 interaction effectively promotes tumor angiogenesis via strong ligand-receptor binding, which also downregulates signaling pathways favoring rapid tumorigenesis. VEGFR-2 activation also appears to correlate with AKT/mTOR signaling [25, 26]. Pharmacological blockade of VEGFR-2 stabilizes endothelial barrier function and suppresses tumor cell extravasation in vivo, emphasizing the importance of VEGFR-2 signaling in tumor invasion and metastasis [27].

VEGFR-2 activation can be inhibited using a number of pharmacodynamic approaches, including receptor blockade (ramucirumab), ligand capture (bevacizumab, also known as avastin), and small-molecule inhibition (sorafenib, sunitinib, apatinib, and cediranib) [28]. The patient described in this report experienced disease progression following gemcitabine-docetaxel chemotherapy. Evidence suggests that some angiogenesis agents can potentiate chemotherapy results in cancer. For example, avastin abrogates reactive resistance, sensitizing both endothelial and cancer cells to therapy by blocking VEGF [29]. Apatinib reverses ABCB1- and ABCG2-mediated multidrug resistance by inhibiting transport, rather than by blocking AKT or ERK1/2 signaling [30]. Although VEGF inhibitors demonstrate potent advantages in cancer therapy, their impacts on overall survival are unclear. Resistance to therapy is associated with highly aggressive cancer phenotypes; therefore, no increase in overall survival has been observed [31].

In 2006, Azizi, et al. were the first to confirm VEGF expression in tumor cells, and VEGFR1 and 2 expression in intra-tumor endothelial cells in a patient with brain metastatic ASPS. The patient was treated with 26 bevacizumab cycles, but experienced disease progression after chemotherapy; eventually, tumor reduction and stable disease were achieved [32]. Other anti-angiogenic therapies have subsequently been used to treat ASPS cases (Table 1). In 2013, a phase II trial of once-daily cediranib, administered in 28-d cycles, was conducted in 43 patients with metastatic, unresectable ASPS, to determine the objective response rate. Sixty percent of patients had stable disease as the best response, with a disease control rate at 24 weeks of 84% [33]. MET is another potential anti-angiogenic target in ASPS treatment, as the ASPL-TFE3 oncoprotein increases MET auto-phosphorylation and activates downstream signaling. However, treatment with tivantinib, a MET inhibitor, only achieved stable disease (no tumor shrinkage) in two ASPS patients [34]. Sunitinib is important in the treatment of advanced or multidrug resistant ASPS. In 2011, nine patients with progressive metastatic ASPS receiving sunitinib (37.5 mg/day) achieved a median progression-free survival time of 17 months [35]. The following year, Abhimanyu, et al. reported a response to sunitinib in an ASPS patient with lung and bone metastases who had not responded to multiple prior chemotherapy regimens [36]. A 2016 retrospective study of 14 unresectable or metastatic ASPS patients treated with sunitinib showed that four patients achieved partial remission, while 10 achieved stable disease [37]. Resistance to anti-angiogenic therapeutics also occurs in ASPS patients. Pazopanib shares many tyrosine kinase targets with sunitinib, including those in the VEGF and platelet-derived growth factor (PDGF) pathways. William, et al. reported on two metastatic ASPS patients for whom sunitinib treatment resulted in stable disease lasting more than one year [38]. Following subsequent disease progression (after sunitinib and second-line bevacizumab), both patients again achieved disease stabilization with pazopanib treatment.

**Table 1:** Anti -angiogenic therapies used in ASPS patients

Time	Treatment Method	Patient Number	Age (years)	Disease Status	Treatment Dose	Clinical Efficacy	Adverse Events
Our study	Apatinib	1	18	Metastatic	500 mg/day	PR PFS: 12-months	Skin rash Liver toxicity Hand-foot syndrome
2016 [37]	Sunitinib	14	14–40	Unresectable or Metastatic	37.5 mg/day	PR: 4 months SD: 10 months Median PFS: 41 months	Bleeding (35.7%) Hair and skin color change (37.5%) Mucositis (28.6%)
2016 [38]	Pazopanib	2	1: 31 2: 26	PD after sunitinib and bevacizuma treatment	1: 800 mg decreased to 600 mg/day 2: 800 mg decreased to 400 mg/day	1: SD: 10 months 2: SD: 8 months	1: Labetalol, diarrhea 2: Hypoxia, pulmonary embolism
2014 [34]	Tivantinib	2	1: 12 2: 15	Metastatic	1: 120–360 mg bid/day 2: 360 mg bid/day	1: SD: 248 weeks 2: PFS: 3 years	1: Hyperbilirubinemia 2: None
2013 [33]	Cediranib	43	19–58	Metastatic Unresectable	30 mg/day	ORR: 35% PR: 15 months SD: 26 months	Hypertension Diarrhea Transaminitis Proteinuria Hypothyroidism
2012 [36]	Sunitinib	1	26	Metastatic	50 mg/day	PR	-
2011 [35]	Sunitinib	9	22–58	Metastatic	37.5 mg/day	PR: 5 months SD: 3 months Progression: 1 months Median PFS: 17 months	Fatigue: 1 Hypothyroidism: 2 Hypertension: 2 Liver toxicity: 1 Nausea and vomit: 1 Neutropenia: 4 Chronic Anemia: 1 Thrombocytopenia: 2
2006 [32]	Bevacizumab	1	8	Metastatic	5–10 mg/kg biweekly	PR	-

PR: partial remission; SD: stable disease; PD: progression disease; PFS: progression free survival; ORR: objective response rate.

The patient in our study has thus far achieved disease-free status for 12 months. Apatinib has produced promising clinical outcomes in several other types of cancer [39–43], and has a high binding affinity relative to other anti-angiogenic drugs. For example, apatinib ligand-receptor binding is 10 times greater than that of sorafenib [44, 45]. Apatinib also binds more strongly to VEGFR-2 than cediranib, a pan-VEGF inhibitor that mainly inhibits VEGFR-1, VEGFR-2, VEGFR-3, and PDGF [46]. Additionally, since bevacizumab, a VEGF antibody, acts by blocking autocrine/paracrine VEGF signaling in tumor cells, intracellular autocrine VEGF signaling in tumor cells can greatly reduce its therapeutic potential. The internal VEGFR-2 inhibitor, apatinib, inhibits intracellular VEGF signaling, suppresses cell proliferation in vitro, and delays xenograft tumor growth in vivo; the anti-VEGF antibody, bevacizumab, demonstrates no such effects [47]. A genome-wide gene expression profile showed that ASPS expresses angiogenic mediators, including VEGF, c-MET, HIF-1α, and angiopoietin-like 2 [48]. Investigation of interactions between these mediators and the ASPS-TEF3 fusion oncoprotein, and exploration of angiogenic mediator inhibitors in combination therapies are warranted.

The tumor size, local resection success, and young age of our patient likely contributed to his survival. The maximum tumor diameter in this case was 4.5 cm. Reports regarding ASPS prognostic factors suggest that tumors smaller than 5 cm are associated with longer progression-free survival [49]. A Surveillance, Epidemiology, and End Results (SEER) Program analysis of ASPS cases demonstrated that large tumor size (> 10 cm) is correlated with poor prognosis [7]. Furthermore, our patient received local tumor resection before chemotherapy and apatinib treatment. For ASPS patients presenting with metastatic disease, surgical resection of the primary tumor is associated with better prognosis [7]. Finally, the patient in our study was only 18 years old. Unlike osteosarcoma and Ewing sarcoma, younger ASPS patients exhibit better 5-year survival than older patients. Children with ASPS have excellent 5-year survival rates of up to 100% [49]; however, the mean 5-year survival for all ASPS is 71% [2] and the mechanisms driving better prognoses in children are not well understood. However, pediatric tumors tend to be smaller than those in adults, with larger tumor size increasing risk of distant metastasis and reduced survival [9].

Although this is a single case report, use of apatinib to treat a hypervascular sarcoma produced a promising and satisfactory clinical outcome. Apatinib may provide an additional treatment option for advanced ASPS, particularly for patients with chemotherapy resistance. However, clinical studies with more cases and longer follow-up times will be required to validate and optimize apatinib use in ASPS patients.

## References

[1] Christopherson WM, Foote FW, Stewart FW (1952). Alveolar soft-part sarcomas; structurally characteristic tumors of uncertain histogenesis. Cancer.

[2] Portera CA, Ho V, Patel SR, Hunt KK, Feig BW, Respondek PM, Yasko AW, Benjamin RS, Pollock RE, Pisters PW (2001). Alveolar soft part sarcoma: clinical course and patterns of metastasis in 70 patients treated at a single institution. Cancer.

[3] Cho YJ, Kim JY (2014). Alveolar soft part sarcoma: clinical presentation, treatment and outcome in a series of 19 patients. Clin Orthop Surg.

[4] Pennacchioli E, Fiore M, Collini P, Radaelli S, Dileo P, Stacchiotti S, Casali PG, Gronchi A (2010). Alveolar soft part sarcoma: clinical presentation, treatment, and outcome in a series of 33 patients at a single institution. Ann Surg Oncol.

[5] Qiao PF, Shen LH, Gao Y, Mi YC, Niu GM (2015). Alveolar soft part sarcoma: Clinicopathological analysis and imaging results. Oncol Lett.

[6] Liu YP, Jin J, Wang WH, Wang SL, Song YW, Fang H, Ren H, Liu XF, Yu ZH, Li YX (2015). A retrospective analysis of lung metastasis in 64 patients with alveolar soft part sarcoma. Clin Transl Oncol.

[7] Wang H, Jacobson A, Harmon DC, Choy E, Hornicek FJ, Raskin KA, Chebib IA, DeLaney TF, Chen YL (2016). Prognostic factors in alveolar soft part sarcoma: A SEER analysis. J Surg Oncol.

[8] Falkenstern-Ge RF, Kimmich M, Wohlleber M, Grabner A, Friedel G, Ott G, Leuschner I, Kohlhaufl M (2013). Lung metastasis of primary alveolar soft-part sarcoma occurring 20 years after initial treatment. Case Rep Oncol Med.

[9] Ogura K, Beppu Y, Chuman H, Yoshida A, Yamamoto N, Sumi M, Kawano H, Kawai A (2012). Alveolar soft part sarcoma: a single-center 26-patient case series and review of the literature. Sarcoma.

[10] Reichardt P, Lindner T, Pink D, Thuss-Patience PC, Kretzschmar A, Dorken B (2003). Chemotherapy in alveolar soft part sarcomas. What do we know?. Eur J Cancer.

[11] Li J, Zhao X, Chen L, Guo H, Lv F, Jia K, Yv K, Wang F, Li C, Qian J, Zheng C, Zuo Y (2010). Safety and pharmacokinetics of novel selective vascular endothelial growth factor receptor-2 inhibitor YN968D1 in patients with advanced malignancies. BMC Cancer.

[12] Ding J, Chen X, Gao Z, Dai X, Li L, Xie C, Jiang H, Zhang L, Zhong D (2013). Metabolism and pharmacokinetics of novel selective vascular endothelial growth factor receptor-2 inhibitor apatinib in humans. Drug Metab Dispos.

[13] Huang L, Wei Y, Shen S, Shi Q, Bai J, Li J, Qin S, Yu H, Chen F Therapeutic effect of apatinib on overall survival is mediated by prolonged progression-free survival in advanced gastric cancer patients. Oncotarget.

[14] Dong M, Bi J, Liu X, Wang B, Wang J (2016). Significant partial response of metastatic intra-abdominal and pelvic round cell liposarcoma to a small-molecule VEGFR-2 tyrosine kinase inhibitor apatinib: A case report. Medicine (Baltimore).

[15] Ji G, Hong L, Yang P (2016). Successful treatment of advanced malignant fibrous histiocytoma of the right forearm with apatinib: a case report. Onco Targets Ther.

[16] Ji G, Hong L, Yang P (2016). Successful treatment of angiosarcoma of the scalp with apatinib: a case report. Onco Targets Ther.

[17] Tsuchida Y, Therasse P (2001). Response evaluation criteria in solid tumors (RECIST): new guidelines. Med Pediatr Oncol.

[18] Ladanyi M, Lui MY, Antonescu CR, Krause-Boehm A, Meindl A, Argani P, Healey JH, Ueda T, Yoshikawa H, Meloni-Ehrig A, Sorensen PH, Mertens F, Mandahl N (2001). The der(17)t(X;17)(p11;q25) of human alveolar soft part sarcoma fuses the TFE3 transcription factor gene to ASPL, a novel gene at 17q25. Oncogene.

[19] Ishiguro N, Yoshida H (2016). ASPL-TFE3 Oncoprotein Regulates Cell Cycle Progression and Induces Cellular Senescence by Up-Regulating p21. Neoplasia.

[20] Carmeliet P, Jain RK (2000). Angiogenesis in cancer and other diseases. Nature.

[21] Folkman J (1971). Tumor angiogenesis: therapeutic implications. N Engl J Med.

[22] Glade-Bender J, Kandel JJ, Yamashiro DJ (2003). VEGF blocking therapy in the treatment of cancer. Expert Opin Biol Ther.

[23] Siveen KS, Prabhu K, Krishnankutty R, Kuttikrishnan S, Tsakou M, Alali FQ, Dermime S, Mohammad RM, Uddin S (2017). Vascular Endothelial Growth Factor (VEGF) Signaling in Tumour Vascularization: Potential and Challenges. Curr Vasc Pharmacol.

[24] Autiero M, Waltenberger J, Communi D, Kranz A, Moons L, Lambrechts D, Kroll J, Plaisance S, De Mol M, Bono F, Kliche S, Fellbrich G, Ballmer-Hofer K (2003). Role of PlGF in the intra- and intermolecular cross talk between the VEGF receptors Flt1 and Flk1. Nat Med.

[25] Trinh XB, Tjalma WA, Vermeulen PB, Van den Eynden G, Van der Auwera I, Van Laere SJ, Helleman J, Berns EM, Dirix LY, van Dam PA (2009). The VEGF pathway and the AKT/mTOR/p70S6K1 signalling pathway in human epithelial ovarian cancer. Br J Cancer.

[26] Peng H, Zhang Q, Li J, Zhang N, Hua Y, Xu L, Deng Y, Lai J, Peng Z, Peng B, Chen M, Peng S, Kuang M (2016). Apatinib inhibits VEGF signaling and promotes apoptosis in intrahepatic cholangiocarcinoma. Oncotarget.

[27] Weis S, Cui J, Barnes L, Cheresh D (2004). Endothelial barrier disruption by VEGF-mediated Src activity potentiates tumor cell extravasation and metastasis. J Cell Biol.

[28] Roviello G, Petrioli R, Marano L, Polom K, Marrelli D, Perrella A, Roviello F (2016). Angiogenesis inhibitors in gastric and gastroesophageal junction cancer. Gastric Cancer.

[29] Blagosklonny MV (2005). How Avastin potentiates chemotherapeutic drugs: action and reaction in antiangiogenic therapy. Cancer Biol Ther.

[30] Mi YJ, Liang YJ, Huang HB, Zhao HY, Wu CP, Wang F, Tao LY, Zhang CZ, Dai CL, Tiwari AK, Ma XX, To KK, Ambudkar SV (2010). Apatinib (YN968D1) reverses multidrug resistance by inhibiting the efflux function of multiple ATP-binding cassette transporters. Cancer Res.

[31] Blagosklonny MV (2005). Why therapeutic response may not prolong the life of a cancer patient: selection for oncogenic resistance. Cell Cycle.

[32] Azizi AA, Haberler C, Czech T, Gupper A, Prayer D, Breitschopf H, Acker T, Slavc I (2006). Vascular-endothelial-growth-factor (VEGF) expression and possible response to angiogenesis inhibitor bevacizumab in metastatic alveolar soft part sarcoma. Lancet Oncol.

[33] Kummar S, Allen D, Monks A, Polley EC, Hose CD, Ivy SP, Turkbey IB, Lawrence S, Kinders RJ, Choyke P, Simon R, Steinberg SM, Doroshow JH (2013). Cediranib for metastatic alveolar soft part sarcoma. J Clin Oncol.

[34] Wagner AJ, Goldberg JM, Dubois SG, Choy E, Rosen L, Pappo A, Geller J, Judson I, Hogg D, Senzer N, Davis IJ, Chai F, Waghorne C (2012). Tivantinib (ARQ 197), a selective inhibitor of MET, in patients with microphthalmia transcription factor-associated tumors: results of a multicenter phase 2 trial. Cancer.

[35] Stacchiotti S, Negri T, Zaffaroni N, Palassini E, Morosi C, Brich S, Conca E, Bozzi F, Cassinelli G, Gronchi A, Casali PG, Pilotti S (2011). Sunitinib in advanced alveolar soft part sarcoma: evidence of a direct antitumor effect. Ann Oncol.

[36] Ghose A, Tariq Z, Veltri S (2012). Treatment of multidrug resistant advanced alveolar soft part sarcoma with sunitinib. Am J Ther.

[37] Li T, Wang L, Wang H, Zhang S, Atikan K, Zhang X, Luo Z, Wang C (2016). A retrospective analysis of 14 consecutive Chinese patients with unresectable or metastatic alveolar soft part sarcoma treated with sunitinib. Invest New Drugs.

[38] Read WL, Williams F (2016). Metastatic Alveolar Soft Part Sarcoma Responsive to Pazopanib after Progression through Sunitinib and Bevacizumab: Two Cases. Case Rep Oncol.

[39] Lin Y, Wang C, Gao W, Cui R, Liang J (2017). Overwhelming rapid metabolic and structural response to apatinib in radioiodine refractory differentiated thyroid cancer. Oncotarget.

[40] Zhang H, Chen F, Wang Z, Wu S (2017). Successful treatment with apatinib for refractory recurrent malignant gliomas: a case series. Onco Targets Ther.

[41] Zhou N, Liu C, Hou H, Zhang C, Liu D, Wang G, Liu K, Zhu J, Lv H, Li T, Zhang X (2016). Response to apatinib in chemotherapy-failed advanced spindle cell breast carcinoma. Oncotarget.

[42] Ding L, Li QJ, You KY, Jiang ZM, Yao HR (2016). The Use of Apatinib in Treating Nonsmall-Cell Lung Cancer: Case Report and Review of Literature. Medicine (Baltimore).

[43] Kou P, Zhang Y, Shao W, Zhu H, Zhang J, Wang H, Kong L, Yu J Significant efficacy and well safety of apatinib in an advanced liver cancer patient: a case report and literature review. Oncotarget.

[44] Tian S, Quan H, Xie C, Guo H, Lu F, Xu Y, Li J, Lou L (2011). YN968D1 is a novel and selective inhibitor of vascular endothelial growth factor receptor-2 tyrosine kinase with potent activity in vitro and in vivo. Cancer Sci.

[45] Mendel DB, Laird AD, Xin X, Louie SG, Christensen JG, Li G, Schreck RE, Abrams TJ, Ngai TJ, Lee LB, Murray LJ, Carver J, Chan E (2003). In vivo antitumor activity of SU11248, a novel tyrosine kinase inhibitor targeting vascular endothelial growth factor and platelet-derived growth factor receptors: determination of a pharmacokinetic/pharmacodynamic relationship. Clin Cancer Res.

[46] Tang W, McCormick A, Li J, Masson E (2016). Clinical Pharmacokinetics and Pharmacodynamics of Cediranib. Clin Pharmacokinet.

[47] Peng S, Zhang Y, Peng H, Ke Z, Xu L, Su T, Tsung A, Tohme S, Huang H, Zhang Q, Lencioni R, Zeng Z, Peng B (2016). Intracellular autocrine VEGF signaling promotes EBDC cell proliferation, which can be inhibited by Apatinib. Cancer Lett.

[48] Stockwin LH, Vistica DT, Kenney S, Schrump DS, Butcher DO, Raffeld M, Shoemaker RH (2009). Gene expression profiling of alveolar soft-part sarcoma (ASPS). BMC Cancer.

[49] Kayton ML, Meyers P, Wexler LH, Gerald WL, LaQuaglia MP (2006). Clinical presentation, treatment, and outcome of alveolar soft part sarcoma in children, adolescents, and young adults. J Pediatr Surg.

